# Comparison of medical resources and costs among patients with coronary heart disease and impaired glucose tolerance in the Acarbose Cardiovascular Evaluation trial

**DOI:** 10.1111/1753-0407.13473

**Published:** 2023-11-01

**Authors:** Liam Mc Morrow, Frauke Becker, Ruth L. Coleman, Hertzel C. Gerstein, Lars Rydén, Stefan Schöder, Alastair M. Gray, Jose Leal, Rury R. Holman, for the ACE Study Group

**Affiliations:** ^1^ Health Economics Research Centre University of Oxford Oxford UK; ^2^ Diabetes Trials Unit, Radcliffe Department of Medicine University of Oxford Oxford UK; ^3^ Department of Medicine and Population Health Research Institute McMaster University and Hamilton Health Sciences Hamilton Canada; ^4^ Department of Medicine Solna Karolinska Institutet Stockholm Sweden; ^5^ Pharma Division Bayer AG Berlin Germany

**Keywords:** acarbose, diabetes, impaired glucose tolerance, prediabetes, resource use, costs

## Abstract

**Background:**

The Acarbose Cardiovascular Evaluation (ACE) trial (ISRCTN91899513) evaluated the alpha‐glucosidase inhibitor acarbose, compared with placebo, in 6522 patients with coronary heart disease and impaired glucose tolerance in China and showed a reduced incidence of diabetes. We assessed the within‐trial medical resource use and costs, and quality‐adjusted life years (QALYs).

**Methods:**

Resource use data were collected prospectively within the ACE trial. Hospitalizations, medications, and outpatient visits were valued using Chinese unit costs. Medication use was measured in drug days, with cardiovascular and diabetes drugs summed across the trial by participant. Health‐related quality of life was captured using the EuroQol‐5 Dimension‐3 Level questionnaire. Regression analyses were used to compare resource use, costs, and QALYs, accounting for regional variation. Costs and QALYs were discounted at 3% yearly.

**Results:**

Hospitalizations were 6% higher in the acarbose arm during the trial (rate ratio 1.06, *p* = .009), but there were no significant differences in total inpatient days (rate ratio 1.04, *p* = .30). Total costs per participant, including study drug, were significantly higher for acarbose (¥ [Yuan] 56 480, £6213), compared with placebo (¥48 079, £5289; mean ratio 1.18, *p* < 0.001). QALYs reported by participants in the acarbose arm (3.96 QALYs) were marginally higher than in the placebo arm (3.95 QALYs), but the difference was not statistically significant (0.01 QALYs; *p* = .58).

**Conclusions:**

Acarbose, compared with placebo, participants cost more due to study drug costs and reported no statistically significant difference in QALYs. These higher within‐trial costs could potentially be offset in future by savings from the acarbose‐related lower incidence of diabetes.

## Graphical abstract

**Highlights**

- The Acarbose Cardiovascular Evaluation trial compared quality adjusted life years (QALYs) and costs of acarbose relative to placebo in participants with coronary heart disease and impaired glucose tolerance in China.
- The acarbose group reported numerically higher QALYs than the placebo group.
- Total costs per participant, including study drug, were significantly higher for the acarbose group compared with placebo.

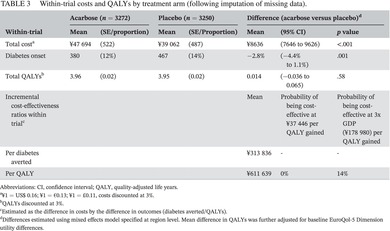

## 1 INTRODUCTION

The global prevalence of diabetes is projected to rise from 425 million in 2017 to 629 million by 2045,^1^ imposing substantial costs on healthcare systems. Diabetes‐related complications comprise a large component of health care costs and are significantly associated with glycemic deterioration.^2^ These costs may be reduced through the early treatment of individuals with “prediabetes,” a condition characterized by impaired fasting glucose, impaired glucose tolerance (IGT), and/or elevated glycated hemoglobin (HbA_1c_) below the threshold for diagnosis of diabetes.^3^ Individuals with prediabetes face an increased risk of developing type 2 diabetes (T2D), as well as cardiovascular disease (CVD), early stage nephropathy, chronic kidney disease, and diabetic retinopathy.^4^ In China, 493 million people are estimated to have prediabetes^5^ and 110 million people (9.7% of the population) currently live with T2D, with the prevalence expected to increase to 140 million by 2040.^6^

The Acarbose Cardiovascular Evaluation (ACE) trial assessed the effects of acarbose, an alpha‐glucosidase inhibitor, in 6522 patients with coronary heart disease (CHD) and IGT from 176 hospital outpatient clinics in China. Acarbose reduces postprandial hyperglycemia by delaying carbohydrate absorption after meals.^7^ The primary objective of the ACE trial was to determine whether, compared with placebo, acarbose therapy when added to optimized cardiovascular therapy in patients with CHD and IGT could reduce cardiovascular‐related morbidity and mortality in a Chinese health care setting. A secondary objective of the ACE trial was to determine if acarbose therapy could prevent or delay new‐onset diabetes in this patient population. Acarbose did not reduce the risk of major adverse cardiovascular events but did reduce diabetes incidence by 18% (*p* = .005).^8^, ^9^

The aim of this study was to compare within‐trial medical resource use and costs between the ACE trial acarbose and placebo arms. Furthermore, we estimated the incremental costs per quality‐adjusted life year (QALY) gained and diabetes averted within the first 6 years of follow‐up.

## 2 METHODS

The ACE trial was an investigator‐sponsored study whose main results and trial design have been reported previously.^8^, ^9^ Eligible patients had IGT diagnosed on a single standard oral glucose tolerance test, defined as a 2‐h plasma glucose value ≥7.8 but <11.1 mmol/L and a fasting plasma glucose (FPG) <7.0 mmol/L within 6 months prior to enrolment. Patients also had established CHD (previous myocardial infarction, previous unstable angina, or current stable angina) and were allocated randomly 1:1 to double‐blind therapy with acarbose (50 mg) or matching placebo taken three times a day with meals. Prior to database lock, the sample size target was revised downwards from 7500 to 6500 as the number of primary events required could be reached with fewer patients.^8^ The protocol was approved by the University of Oxford Tropical Research Ethics Committee and by central or local ethics committees (as appropriate) at participating sites. All participants provided written informed consent.

The primary composite cardiovascular outcome was a five‐point major adverse cardiovascular event defined as cardiovascular death, nonfatal myocardial infarction, nonfatal stroke, hospital admission for unstable angina, or hospital admission for heart failure. Secondary outcomes included resource use, costs, health utility, and cost‐effectiveness.

### 2.1 Economic analysis

The academic health economics team from the University of Oxford led the analysis presented in this study. The health economic analysis plan was finalized prior to database lock. Medical resource use data (hospitalizations, medications, outpatient visits and study drug) were collected throughout the trial, unit cost data were gathered from secondary sources. Data were truncated at the end of year six of follow‐up due to attrition.

#### 2.1.1 Medical resource use and cost assignment

Hospitalization data, recorded on an electronic case report forms (eCRF), included the number of inpatient days and the reason for admission. The 2016 China Health and Family Planning Statistical Yearbook provided unit cost data on the in‐hospital cost per day, by reason for admission^10^ (see Table S1). These unit costs were multiplied by the number of days in hospital within the trial.

Hospital outpatient visits in the previous 12 months were self‐reported by the trial participant at each annual study visit, but the eCRF did not ask participants to differentiate between study visits and outpatient visits. Therefore, when the number of self‐reported outpatient visits was lower than the number of recorded study visits, the latter was used. Outpatient visit costs were obtained by multiplying the unit cost of an outpatient visit^11^ (see Table S1) by the number of visits.

At each annual visit, trial participants were asked to report whether they were taking any of 24 classes of cardiovascular medications and any of 10 classes of diabetes medications. The participant's response was assumed to apply to the entire year, allowing the calculation of annual drug days. If the participant reported starting or stopping a drug, the change was assumed to have occurred at the midpoint of the preceding year. Drug days for cardiovascular drugs and diabetes drugs were summed within each year to calculate cardiovascular and diabetes drug days. Total drug days were summed across cardiovascular drug days and diabetes drug days. Drug unit costs were obtained from the Beijing Medicine Sunshine Purchase Platform^12^ and published studies^11^, ^13^ (see Table S1). The unit costs were converted into a daily cost per medication by dividing the respective package size/quantity by the World Health Organization (WHO) Defined Daily Dose.^14^ The daily unit cost was then multiplied by the number of drug days to calculate the annual medication cost.

The study drug was dispensed to participants at each 4 month visits. Participants in the placebo arm were assumed not to take the active study drug. An acarbose dose of 50 mg three times daily, as stated in study protocol,^9^ was used to calculate a cost per drug day. If participants did not attend the study visit they did not receive acarbose and, therefore, did not take it for the 4 months following the study visit.

We applied a discount rate of 3% for costs and outcomes after the first year of follow‐up, consistent with WHO guidelines for discounting in cost‐effectiveness analysis.^15^ Where appropriate, costs were inflated to 2017 prices using the World Bank Consumer Price Index.^16^

#### 2.1.2 Quality‐adjusted life years and diabetes onset

Health‐related quality of life was captured using the EuroQol‐5 Dimension‐3 Level (EQ‐5D‐3L) questionnaire.^17^ This was administered to participants at baseline and each annual visit, and occasionally during 4‐monthly visits. Responses to the EQ‐5D‐3L questionnaire were converted into health utilities using a Chinese value set.^18^ The difference in EQ‐5D utility was 0.002 (95% confidence interval [CI], −0.002 to 0.007, *p* = .278) between the acarbose and placebo groups during their follow‐up period. The methods and results of the EQ‐5D utility analysis were published previously.^19^ QALYs were calculated using the area under the curve approach, which involves estimating the average EQ‐5D utility between each follow‐up time and weighting it by survival time. Partially completed EQ‐5D‐3L questionnaires were considered missing.

#### 2.1.3 Missing data

Missing data for inpatient days were imputed using the mean values by reason for admission and region since hospital admission data were complete. As cardiovascular and diabetes medications reported within the eCRF were typically used over long periods of time by the participant, missing cardiovascular and diabetes medication data were imputed by carrying the last value forward. Study drug data were complete. Missing outpatient and EQ‐5D utility data were imputed following examination of the patterns of missingness with multiple imputation being used, if data were missing at random (MAR) was a plausible assumption.^20^ Accordingly, we checked the proportion of missing data by allocated treatment group at different follow‐up time points, with an imbalance suggesting a departure from MAR. Furthermore, using logistic regressions, we examined whether the probability of data missing at each follow‐up period was (a) associated with baseline covariates (allocated treatment, sex, body mass index, HbA_1c_, FPG, age, and EQ‐5D); and (b) associated with previously observed data (eg, missing outpatient data regressed on observed outpatient visits in the prior 1–4 years). A significant association between missing data and both baseline covariates and previously observed values suggests MAR as a plausible assumption to conduct the cost‐effectiveness analysis. Details of methods used for multiple imputation of outpatient and EQ‐5D utility values are provided in the Appendix.

#### 2.1.4 Within‐trial analysis

Descriptive statistics, including sample size and means and SDs of medical resource use and costs were reported by treatment group and by year. Differences between treatment groups in medical resource use and costs were reported as rate ratios and mean ratios respectively, with bootstrapped 95% CIs.

Following imputation, multilevel generalized linear mixed models (GLMM) were used to compare counts of medical resource use and costs between treatment groups.^21^ Resource use models used negative binomial distributions and log links whereas cost models used gamma distributions and log links. Each multilevel GLMM specified the treatment assignment as a fixed effect and patients (level 1) were clustered within regions (level 2) and cluster‐robust standard errors (*meglm* in STATA). For multiple imputed data, estimates derived from each imputed data set were combined using Rubin's rule. Rate ratios are reported in resource use models and mean ratios are reported in cost models, whereby a value greater than one indicates a higher average count or average cost in the treatment arm, while holding all other variables in the model constant. Sensitivity analyses explored the impact on the medical resource use and cost results of using only observed data and of varying discount rates for costs (1% and 5%).

We also estimated the incremental cost per diabetes onset averted and the incremental cost by QALY saved within the 6 years of follow‐up. To account for multiple imputation and capture the correlation between incremental cost and outcomes, we bootstrapped 1000 times from each of the imputed data sets, ran the estimation models on each bootstrapped data set, and extracted the estimated treatment effects. The estimation models for total QALYs and costs consisted of multilevel mixed effects linear regressions controlling for treatment allocation and, for QALYs, baseline EQ‐5D utility, with robust SEs and regions modeled as random intercepts (*mixed* in STATA). We judged acarbose to be cost effective adopting the cost‐effectiveness thresholds of ¥37446^22^ and ¥178 980 (three times the gross domestic product per capita of China in 2017^23^). All analyses were run in STATA version 17.0.

## 3 RESULTS

Of the 6522 patients recruited to the trial, 3272 were allocated to acarbose and 3250 to the placebo arm and followed up for a median of 5.0 years (interquartile range 3.4–6.0) in both groups. The characteristics of the groups were well balanced at baseline. The mean age at baseline was 64.3 years (SD 8.1) and 4760 (73%) were male. The mean EQ‐5D utility at baseline was 0.93 (SD 0.10).

Across all observations for the first 6 years of follow‐up, 35% and 38% of outpatient visit data and EQ‐5D utility data were missing, respectively (Table S2). The proportion of participants with missing data increased with the duration of follow‐up but remained similar between treatment groups. The vast majority of missing data were due to participants who were followed up for the primary end point but did not attend annual follow‐up visits. Table S3 reports the odds ratios for missing visit and EQ‐5D data for a selection of baseline variables. EQ‐5D at baseline, sex, body mass index, and age were significantly associated with missingness. Missingness was also significantly associated with previous observed visits and EQ‐5D data (Table S4). Hence, the patterns of missingness were suggestive that the data were MAR was a plausible assumption. Therefore, missing data for outpatient visits and EQ‐5D utility were imputed using multiple imputation.

### 3.1 Resource use and costs

Over the 6 years of follow‐up, the mean number of hospitalizations (0.5, SD 1.1) in the acarbose arm was significantly higher than in the placebo arm (0.4, SD 1.0) (rate ratio 1.06, *p* = .009), albeit the magnitude of the difference was small. There were no significant differences in inpatient days, outpatient visits, cardiovascular drug days, or total drug days between treatment arms within the trial follow‐up period (Table 1). The average numbers of inpatient days and outpatient visits during the trial follow‐up period were similar across the treatment (means of 5.0 days and 51.7 visits) and placebo arm (means of 4.8 days and 51.2 visits) (inpatient days: rate ratio 1.04, *p* = .30; outpatient visits: rate ratio 1.01, *p* = .54). Cardiovascular drug days (rate ratio 1.0, *p* = .73) and total drug days (rate ratio 0.99, *p* = 0.60) were also similar across trial arms. Mean diabetes drug days were significantly lower in the acarbose arm (73 days) compared with the placebo arm (95 days) with a rate ratio of 0.80 (*p* = .043). Descriptive results of medical resource use are presented in Table S5.

**TABLE 1:** Within‐trial resource use by treatment arm (following imputation of missing data).

Resource use	Acarbose (*n* = 3272)		Placebo (*n* = 3250)		Difference (acarbose versus placebo)^b^		
	Mean	(SD)	Mean	(SD)	Rate ratio^c^	(95% CI)	*p* value
Hospitalizations	0.5	(1.1)	0.4	(1.0)	1.06	(1.02–1.11)	.009
Inpatient days	5.0	(14.1)	4.8	(12.8)	1.04	(0.97–1.12)	.29
Outpatient visits	51.7	(53.0)	51.2	(53.3)	1.01	(0.98–1.04)	.54
Diabetes drug days^a^	73	(334.4)	95	(339.0)	0.80	(0.60–0.99)	.04
CV drug days^a^	6142	(2987.3)	6174	(2995)	1.00	(0.97–1.03)	.73
Total drug days^a^	6215	(3034.9)	6269	(3044.1)	0.99	(0.96–1.02)	.60
Study drug days	1468	(626)	‐	‐	‐	‐	‐

Abbreviations: CI, confidence interval; CV, cardiovascular.

^a^ Represents the number of drugs per day summed across the follow‐up period for each patient excluding study drug.

^b^ Differences estimated using Stata MEGLM (multilevel mixed‐effects generalized linear model) procedure specified at region levels with log‐link function and negative binomial distribution.

^c^ A rate ratio >1 indicates a higher average count in the treatment group relative to placebo group, for example being in the treatment group increases the expected number of hospitalizations by 6% relative to the placebo group.

Costs over the trial period for inpatient care, outpatient care, diabetes medications, cardiovascular medications, total medications, and total costs (excluding study drug) did not differ significantly between groups (Table 2). Despite lower diabetes drug days in the acarbose arm, no significant differences in diabetes medication costs were observed (acarbose: ¥257, placebo: ¥307, mean ratio 0.84, *p* = .23). On average, study drug costs were ¥8512 (€1107; £936; $1362) per patient in the acarbose arm within trial. The total cost per patient allocated to the acarbose group (¥47 694) was significantly higher than the placebo group (¥39 062) at ¥8512 (€1107, £936, $1362, mean ratio 1.23, *p* < .001). Descriptive results of costs by year are presented in Table S5. The sensitivity analyses show that the results were relatively robust to using only the observed data (Table S6) and to varying the discount rate for costs between 1% and 5% (Table S7).

**TABLE 2:** Within‐trial costs by treatment arm (following imputation of missing data).

Chinese Yuan (2017)^a^	Acarbose (*n* = 3272)		Placebo (*n* = 3250)		Difference (acarbose versus placebo)^c^		
	Mean	(SD)	Mean	(SD)	Mean ratio^d^	(95% CI)	*p* value
Inpatient care cost	¥5316	(14790)	¥5218	(13602)	1.02	(0.96–1.08)	.60
Outpatient care cost	¥16 512	(17460)	¥16 342	(17369)	1.01	(0.98–1.04)	.49
Diabetes drug cost^b^	¥257	(2349)	¥307	(1835)	0.84	(0.62–1.12)	.23
CV drug cost	¥17 096	(9665)	¥17 196	(9798)	0.99	(0.98–1.01)	.30
Total drugs cost^b^	¥17 354	(10191)	¥17 503	(9998)	0.99	(0.96–1.03)	.60
Study drug cost	¥8512	(3558)	‐	‐	‐	‐	‐
Total cost^b^	¥39 182	(27741)	¥39 062	(27741)	1.00	(0.98–1.03)	.89
Total cost	¥47 694	(29870)	¥39 062	(27741)	1.23	(1.19–1.27)	<.001

Abbreviations: CI, confidence interval; CV, cardiovascular.

^a^ ¥1 = US$ 0.16; ¥1 = €0.13; ¥1 = £0.11, costs discounted at 3%.

^b^ Cost per patient excluding study drug.

^c^ Differences estimated using Stata MEGLM (multilevel mixed‐effects generalized linear model) procedure specified at region level with log‐link function and gamma distribution.

^d^ A mean ratio >1 indicates a higher average cost in the treatment group relative to the placebo group, for example, being in the treatment group increases the hospitalization costs by 2% relative to the placebo group.

### 3.2 Diabetes onset and QALYs

Table 3 reports the outcomes and total costs in both arms of the trial. The number of participants diagnosed with diabetes over the first 6 years of the trial was significantly lower in the acarbose arm (*n* = 380) compared with the placebo arm (*n* = 467) (*p* = .005).^8^ Hence, over 6 years of follow‐up, 2.8% (95% CI, 1.1%–4.4%) of diabetes cases were averted in the acarbose arm compared with the placebo arm. Participants in the acarbose arm also reported higher QALYs (3.96 QALYs) compared with the placebo arm (3.95 QALYs), but the difference was not statistically significant (0.014 QALYs; *p* = .58).

**TABLE 3:** Within‐trial costs and QALYs by treatment arm (following imputation of missing data).

Within‐trial	Acarbose (*n* = 3272)		Placebo (*n* = 3250)		Difference (acarbose versus placebo)^d^		
	Mean	(SE/proportion)	Mean	(SE/Proportion)	Mean	(95% CI)	*p* value
Total cost^a^	¥47 694	(522)	¥39 062	(487)	¥8636	(7646 to 9626)	<.001
Diabetes onset	380	(12%)	467	(14%)	−2.8%	(−4.4% to 1.1%)	.001
Total QALYs^b^	3.96	(0.02)	3.95	(0.02)	0.014	(−0.036 to 0.065)	.58

Incremental cost‐effectiveness ratios within trial^c^					Mean	Probability of being cost‐effective at ¥37 446 per QALY gained	Probability of being cost‐effective at 3x GDP (¥178 980) per QALY gained
Per diabetes averted					¥313 836	‐	‐
Per QALY					¥611 639	0%	14%

Abbreviations: CI, confidence interval; QALY, quality‐adjusted life years.

^a^ ¥1 = US$ 0.16; ¥1 = €0.13; ¥1 = £0.11, costs discounted at 3%.

^b^ QALYs discounted at 3%.

^c^ Estimated as the difference in costs by the difference in outcomes (diabetes averted/QALYs).

^d^ Differences estimated using mixed effects model specified at region level. Mean difference in QALYs was further adjusted for baseline EuroQol‐5 Dimension utility differences.

The incremental cost per diagnosis of diabetes averted of acarbose relative to placebo was estimated as ¥313 836 (£34 522, €40 799). Figure S1 shows the cost‐effectiveness scatter plot giving differences in mean total costs and diabetes cases averted. Most bootstrap replicates remained in the northeast quadrant of the cost‐effectiveness plot, indicating that acarbose resulted in higher number of diabetes cases averted but also higher costs relative to the placebo arm.

**FIGURE 1:**
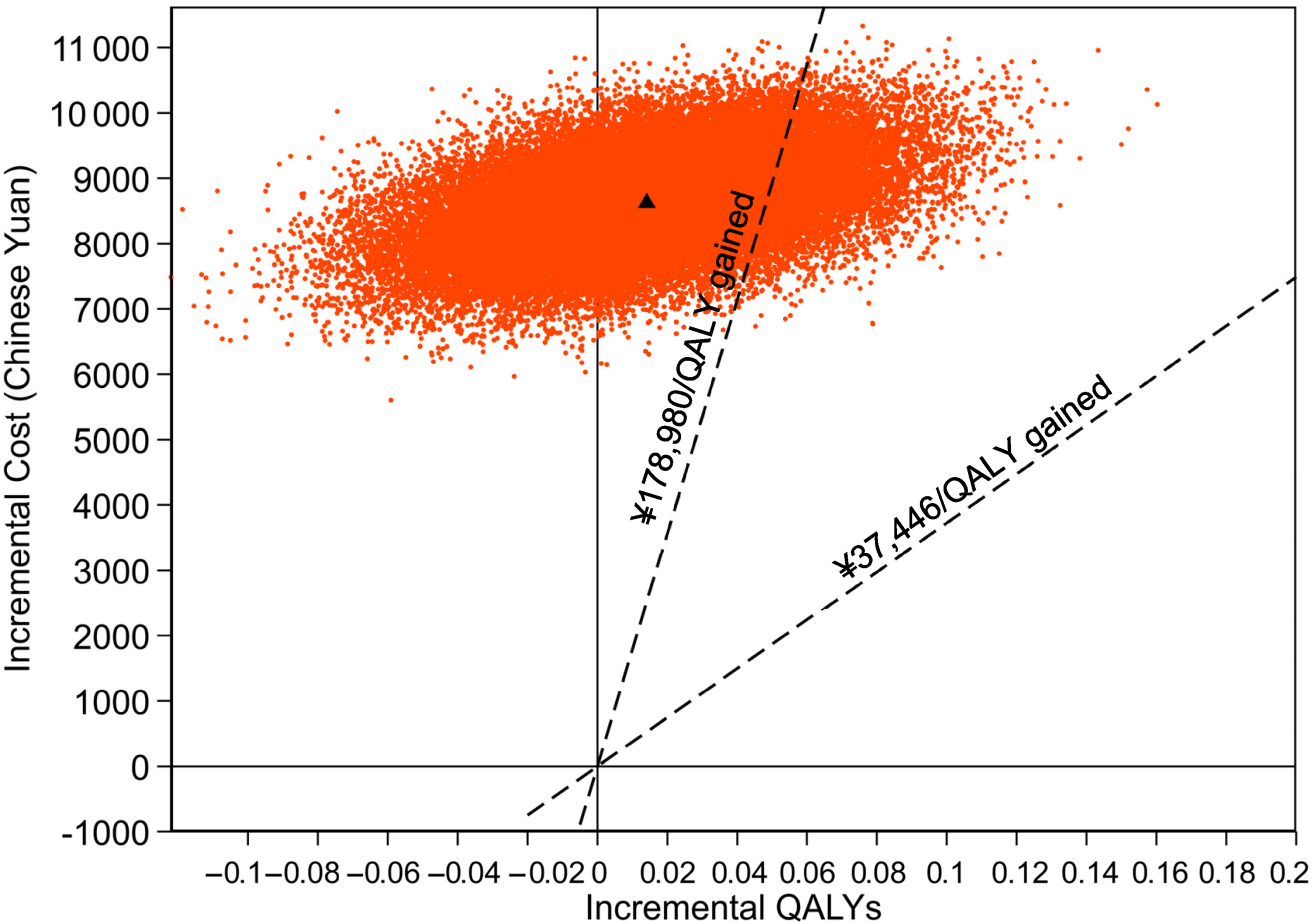
Cost‐effectiveness scatter plot (within‐trial). Scatter plot of estimated joint density of incremental costs and QALYs of acarbose relative to placebo obtained by bootstrap resampling from each of the imputed data sets, running the regression models on each bootstrapped data set, and extracting the estimated incremental costs and QALYs gained. The black triangle represents the estimated mean incremental costs and QALYs. Dashed lines represent threshold values of ¥37446^22^ and ¥178 980 (3× GDP per capita^23^) per QALY gained. Bootstrapped results falling below the lines are deemed cost effective. From the bootstrapped results, we calculated the probability that acarbose was more cost‐effective than placebo for the two different threshold values per QALY gained (Table 3). GDP, gross domestic product; QALY, quality‐adjusted life year.

The incremental cost per QALY gained for acarbose relative to placebo was ¥611 639 (€79 513; £67 280). Figure 1 presents the cost‐effectiveness scatter plot giving differences in mean total costs and QALYs for acarbose versus placebo. Acarbose has higher costs relative to placebo but with considerable uncertainty concerning the gains in QALYs. Over the 6 years of follow‐up, the probability that acarbose treatment is cost effective is 14% at a threshold value of ¥178 980 per QALY (three times the gross domestic product per capita of China in 2017).

## 4 CONCLUSIONS

The ACE trial set out to determine whether acarbose could reduce cardiovascular‐related morbidity and mortality in patients with IGT and CHD. Acarbose, given in addition to optimized cardiovascular therapy, did not reduce the risk of major adverse cardiovascular event but did reduce the incidence of diabetes by 18%.^8^

Our health economic findings are consistent with the ACE trial results. We found no significant differences in medical resource use in terms of inpatient days, cardiovascular drug days, or total drug days between treatment arms, whereas the number of hospitalizations was significantly higher in the acarbose arm and the number of diabetes drug days was significantly lower. The higher hospitalization rate in the acarbose group did not significantly increase inpatient days or hospitalization costs relative to the placebo arm. Diabetes drug days were significantly lower in the acarbose arm due to the lower incidence of diabetes, but this did not translate into significantly lower costs in the acarbose group.

Overall, our analysis found no significant differences in total costs (excluding study drug) during the first 6 years of the trial period across treatment arms. Only after including study drug costs was the acarbose arm significantly more expensive than the placebo arm. However, a significant cost reduction could become evident over an extended time period, as patients with diabetes require more expensive medications such as insulin and are more likely to develop costly complications,^24^ which may in turn reduce health‐related quality of life.^25^

Over the 6 years of follow‐up in the ACE trial, we found acarbose resulting in marginally higher QALYs than placebo, albeit not a significant difference. This may reflect the ceiling effect observed when measuring health‐related quality of life in Chinese populations^26^, ^27^ resulting in low variation in EQ‐5D utility scores observed over time and between trial arms. This is consistent with our previous results reporting health utilities not to differ between arms over time.^19^ Adopting a cost‐effectiveness threshold of three times the gross domestic product per capita, acarbose was not cost effective relative to placebo. However, the trial follow‐up period is clearly insufficient to estimate the cost‐effectiveness of acarbose as it requires an estimation of its impact over the lifetime of individuals. The difference in diabetes onset relative to placebo is likely to have significant medium and long‐term impact on survival, costs, and health‐related quality of life.

Previous within‐trial analyses from the German,^28^ Swedish^29^ and Spanish health care perspective^30^ have reported acarbose treatment to be effective and cost effective or even cost saving, in individuals with impaired glucose tolerance and at high risk of CVD. However, all these analyses were based on data from the Study to Prevent Noninsulin Dependent Diabetes Mellitus (STOP‐NIDDM) trial over 3.3 years, with the costs of acarbose being offset due to the reduced probability of clinical events. By contrast, our study found that acarbose treatment costs were not offset by a reduction in health care resource use in China but rather increased the costs for those with IGT and CHD.

Effective lifestyle interventions remain the recommended strategy for individuals with prediabetes.^31^, ^32^ Glucose‐lowering drugs other than acarbose, such as metformin, have been assessed in randomized trials in people with prediabetes, albeit without a history of cardiovascular disease.^33^ For example, the Diabetes Prevention Program (DPP) showed metformin to reduce diabetes incidence by 31% relative to placebo, compared with the 18% reduction seen with acarbose in ACE, and to accrue 0.02 more QALYs,^34^, ^35^ compared with 0.01 QALYs in our study. Furthermore, a DPP 10‐year cost‐effectiveness analysis showed metformin to be marginally cost saving compared with placebo.^35^ In short‐term trials, other medications such as glucagon‐like peptide‐1 receptor agonists and thiazolidinediones have been shown to decrease progression to diabetes when added to lifestyle interventions.^33^ However, results from cardiovascular outcome trials, impact on QALYs, and the cost‐effectiveness associated with these medications in populations with prediabetes are not yet available. Accordingly, concerns about their long‐term effectiveness and safety profile in prediabetes, given the existence of long‐term safety data for metformin, means that metformin remains the preferred pharmacotherapy option to support lifestyle interventions by organizations such as the American Diabetes Association.^32^ Sodium‐glucose cotransporter 2 inhibitors, with their cardiovascular and renal protective effects, have shown potential in prediabetes with a risk reduction for new‐onset diabetes similar to acarbose in populations with heart failure.^36^ However, the effectiveness of alternative glucose‐lowering drugs in prediabetes populations with CHD such as those in ACE, their additional costs, potential side effects, and duration of efficacy require further consideration.

Conducting an economic evaluation in a Chinese setting raised methodological challenges. National costs were applied to hospitalizations and Beijing‐specific costs were applied to medications due to the lack of available regional cost data. Hence, we were unable to account for regional differences in health care costs across China. There was a sizeable amount of missing data on outpatient visits and EQ‐5D utilities that was accounted for by using multiple imputation. This assumes data are MAR conditional on modeled covariates and we found no strong evidence against this assumption. However, the overall results and conclusions did not differ using alternative imputation methods and in sensitivity analyses. Finally, our analysis also identified some discrepancies between recorded hospitalizations and the number of adjudicated events (excluding deaths) that were captured over the trial period. Because both data sources overlapped for the majority of events (over 90% of overlap), only hospitalization data were used for the analysis reported here. This approach did not require any additional assumptions but may have disregarded, for example, deaths leading to a hospitalization that were subsequently not recorded (about 22% of deaths were associated with a hospitalization). However, evidence exists that the proportion of deaths occurring outside of hospital may vary greatly by age, gender, socioeconomic status, and region in China (ie, 7% to 88%).^37^, ^38^

Future work will aim to calculate the lifetime costs and QALYs associated with acarbose treatment for people with IGT and CVD, taking account of the significant effect of acarbose therapy in preventing or delaying new‐onset T2D. This work will be undertaken by using ACE trial data to build a prediabetes model that simulates progression to diabetes, whereupon the participant will then enter an existing type 2 diabetes model, such as the UK Prospective Diabetes Study Outcomes Model.^39^

This study provides valuable insights into the impact of acarbose on medical resource use and costs in a Chinese setting. Despite the higher costs in the acarbose arm, due to the study drug, there may be offsetting savings over time arising from a reduction in diabetes medications and related complications. Future research is needed to explore the impact of acarbose on resource use, costs, and quality‐adjusted survival over the lifetime horizon.

## AUTHOR CONTRIBUTIONS

All authors commented on previous versions of the manuscript and read and approved the final version. Liam Mc Morrow and Frauke Becker were responsible for the data analysis, interpretation of results, drafting, and critical revision of the manuscript. Alastair M. Gray and Rury R. Holman were responsible for the study concept and design, interpretation of results, drafting, and critical revision of the manuscript. Jose Leal was responsible for the study concept and design, methodology, data analysis, interpretation of results, drafting, and critical revision of the manuscript. Jose Leal had full access to all of the data in the study and is the guarantor of this work.

## CONFLICT OF INTEREST STATEMENT

Rury R. Holman reports research support from AstraZeneca, Bayer (related to the present study), and Merck Sharp & Dohme, and personal fees from Anji Pharmaceuticals, AstraZeneca, Novartis, and Novo Nordisk. Hertzel C. Gerstein reports personal fees from the University of Oxford (related to the present study); grants and personal fees from Sanofi, Eli Lilly, AstraZeneca, Boehringer Ingelheim, Novo Nordisk, and Merck Sharp & Dohme; and personal fees from Abbott and Amgen. Lars Rydén reports grants from Swedish Heart Lung Foundation Swedish Diabetes Foundation, Family E Perssons Foundation, and Amgen; and grants and personal fees from Bayer AG (related to the present study), Boehringer Ingelheim, Merck Sharp & Dohme, and Novo Nordisk. Stefan Schöder was a Bayer AG employee. Frauke Becker, Liam Mc Morrow, Jose Leal, Ruth L. Coleman, and Alastair M. Gray report no conflicts of interest.

## Supporting information


**Data S1.** Supporting Information.
