# Supplementary material for: Comparison of medical resources and costs among patients with coronary heart disease and impaired glucose tolerance in the Acarbose Cardiovascular Evaluation trial
Source: J Diabetes. 2023 Nov 1;16(2):e13473. doi: 10.1111/1753-0407.13473 (PMC10859317; doi:10.1111/1753-0407.13473)
Supplement: Supplementary file 1 — Data S1. Supporting Information. [file JDB-16-e13473-s001.docx]

# Supplementary Appendix

We report here additional results of our manuscript. The order of sections, tables and figures follows their citation in the manuscript. For convenience, we list below the page number of each Figure and Table.

[**Table S1**: Unit Cost Sources 3](#_Toc136600394)

[**Multiple imputation of missing outpatient visit and EQ-5D utility data** 4](#_Toc136600395)

[**Table S2:** Missing EQ-5D utility and outpatient visit data 5](#_Toc136600396)

[**Table S3:** Logistic regression for missingness of outpatient visits and EQ-5D utility on selection of baseline variables* 6](#_Toc136600397)

[**Table S4:** Logistic regression for missingness of outpatient visits and EQ-5D utility previous observed values***** 6](#_Toc136600398)

[**Table S5:** Within Trial Resource Use by Year (following imputation of missing data) 7](#_Toc136600399)

[**Table S5:** Within Trial Costs by Year (following imputation of missing data) 9](#_Toc136600400)

[**Table S6:** Within Trial Costs by Year (using only observed data) 11](#_Toc136600401)

[**Table S7:** Sensitivity Analysis on between group differences in within trial costs when varying discount rates between 1% and 5% (following imputation of missing data) 13](#_Toc136600402)

[**Figure S1**: Cost-effectiveness scatter plot (within trial) 14](#_Toc136600403)

[
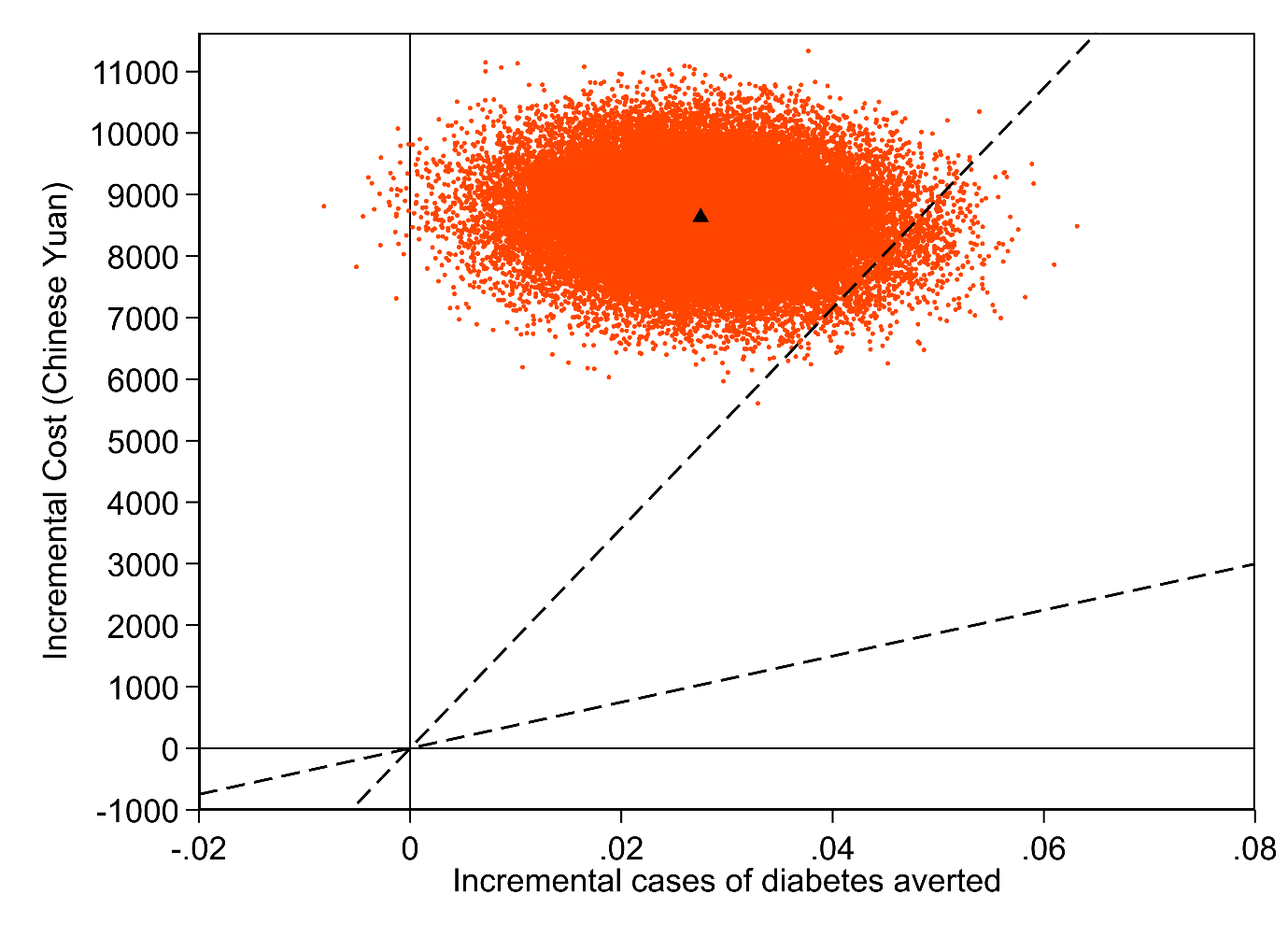
 14](#_Toc136600404)

## **Table S1**: Unit Cost Sources

| **Resource Use** | **Cost per day**  **in Chinese Yuan (2017)^a^** | **Source** |
| --- | --- | --- |
| Acute myocardial infarction | 2771.74 | China Health and Family Planning Statistical Yearbook 2016 ^10^ |
| Unstable angina | 1600.81^b^ |  |
| Stroke/TIA | 849.09^c^ |  |
| Heart failure | 909.41 |  |
| Cardiovascular procedure/operation | 897.02^d^ |  |
| Other cardiovascular | 807.92 |  |
| Cancer | 1292.01 |  |
| Infection | 608.08 |  |
| Other non-cardiovascular | 884.36 |  |
| Outpatient Visits | 364.49^e^ | Wang 2009 ^11^ |
| Biguanide | 2.76 | Beijing Medicine Sunshine Purchase Platform ^12^ |
| Sulfonylurea | 1.32 |  |
| Glinides | 6.74 |  |
| GLP-1 Analogue | 38.87 |  |
| Alpha-glucosidase Inhibitor | 4.15 |  |
| DPP-4 inhibitor | 8.01 |  |
| Short acting insulin | 7.25 |  |
| Long acting insulin | 20.73 |  |
| Mixed insulin | 7.65 |  |
| Other anti-diabetic therapy | 3.17 (TZDs only) |  |
| Statin | 4.75 | Beijing Medicine Sunshine Purchase Platform^12^ (except Eplerinone^13^) |
| Fibrate | 2.87 |  |
| Nicotinic Acid | 5.49 |  |
| Digitalis | 0.93 |  |
| Antiarrhythmic | 8.14 |  |
| Eplerinone | 16.62 |  |
| Spironolactone | 0.69 |  |
| ACE Inhibitors | 1.93 |  |
| Angiotensin Receptor Blocker | 3.05 |  |
| Beta Blockers | 2.8 8 |  |
| Calcium Channel Blockers | 2.01 |  |
| Renin Inhibitors | 10.15 |  |
| Alpha blockers | 2.46 |  |
| Thiazide diuretic | 0.03 |  |
| Non-thiazide diuretic | 0.64 |  |
| Other anti-hypertensive therapy | 2.91 |  |
| Aspirin | 0.30 |  |
| Clopidogrel | 9.75 |  |
| Other anti-platelet therapy | 5.02 |  |
| Nitrates | 1.17 |  |
| Other anti anginals | 1.17 |  |
| Steroids Oral | 0.35 |  |
| Steroids Injections | 3.65 |  |
| Steroids Other (e.g. cream) | 0.54 |  |

^a^ ¥1 = US$0.16; ¥1 = €0.13; ¥1 = £0.11.

^b^ Includes all Angina.

^c^ Translated as cerebrovascular disease.

^d^ Average cost of all cardiovascular diseases.

^e^ Cost data were taken from a cross sectional survey carried out in four major Chinese cities (Beijing, Guangzhou, Shanghai, Chengdu) from March 2007 to September 2007. Annual direct medical costs were estimated based on payment amounts for outpatient services.

## **Multiple imputation of missing outpatient visit and EQ-5D utility data**

Missing outpatient visit and EQ-5D utility score data was imputed for each annual follow-up time point by chained equations as a function of follow-up time, sex, age, ethnicity, baseline risk factors (HbA1c, body mass index, HDL-cholesterol, LDL- cholesterol, smoking status, and others), baseline history of cardiovascular disease, region and recruitment site, and the occurrence of non-fatal cardiovascular events (stroke, myocardial infarction, hospitalisation due to heart failure, and angina), gastrointestinal and diabetes onset during follow-up. The imputation model was run separately by randomised treatment. We then used predicted mean matching to created the imputed datasets with ten nearest neighbours. In periods where death was observed these were adjusted. For outpatient visits and medications, we assumed they were incurred linearly over time, such that, if an individual died 6 months into an annual period, they incurred half the predicted costs and medications. For EQ-5D utility, we assumed the imputed utility score prevailed until the time of death.

## **Table S2:** Missing EQ-5D utility and outpatient visit data

|  | **Acarbose** | | | **Placebo** | | |
| --- | --- | --- | --- | --- | --- | --- |
|  | n | Missing | % | n | Missing | % |
| **EQ-5D utility** | | | | | | |
| 12 months | 3,272 | 870 | 27% | 3,250 | 920 | 28% |
| 24 months | 3,190 | 1,218 | 38% | 3,156 | 1226 | 39% |
| 36 months | 2,923 | 1,215 | 42% | 2,912 | 1287 | 44% |
| 48 months | 2,666 | 1,377 | 52% | 2,644 | 1365 | 52% |
| 60 months | 2,234 | 1,316 | 59% | 2,202 | 1316 | 60% |
| 72 months | 1,623 | 1,145 | 71% | 1,604 | 1120 | 70% |
| Total | 19,180 | 7,141 | 37% | 19,018 | 7,234 | 38% |
| **Outpatient Visits** | | | | | | |
| 12 months | 3,272 | 973 | 30% | 3,250 | 1,021 | 31% |
| 24 months | 3,190 | 1,122 | 35% | 3,156 | 1,124 | 36% |
| 36 months | 2,923 | 1,142 | 39% | 2,912 | 1,207 | 41% |
| 48 months | 2,666 | 1,177 | 44% | 2,644 | 1,182 | 45% |
| 60 months | 2,234 | 1,064 | 48% | 2,202 | 1,060 | 48% |
| 72 months | 1,623 | 806 | 50% | 1,604 | 794 | 50% |
| Total | 19,180 | 6,594 | 34% | 19,018 | 6,688 | 35% |
| **EQ-5D utility or outpatient visits** | | | | | | |
| 12 months | 3,272 | 1,034 | 32% | 3,250 | 1,084 | 33% |
| 24 months | 3,190 | 1,328 | 42% | 3,156 | 1,331 | 42% |
| 36 months | 2,923 | 1,325 | 45% | 2,912 | 1,384 | 48% |
| 48 months | 2,666 | 1,447 | 54% | 2,644 | 1,427 | 54% |
| 60 months | 2,234 | 1,380 | 62% | 2,202 | 1,368 | 62% |
| 72 months | 1,623 | 1,170 | 72% | 1,604 | 1,147 | 72% |
| Total | 19,180 | 7,994 | 42% | 19,018 | 8,041 | 42% |

## **Table S3:** Logistic regression for missingness of outpatient visits and EQ-5D utility on selection of baseline variables*

|  | **Missing data on outpatient visits** | **Missing data on EQ-5D** |
| --- | --- | --- |
| **Baseline variables** | **Odds ratio (p-value)** | **Odds ratio (p-value)** |
| Treatment allocation | 0.95 (0.262) | 0.96 (0.233) |
| Female | 1.25 (<0.001) | 1.28 (<0.001) |
| BMI | 0.95 (<0.001) | 0.97 (<0.001) |
| Age | 1.03 (<0.001) | 1.02 (<0.001) |
| HbA1c | 1.04 (0.309) | 1.00 (0.915) |
| EQ-5D | 0.49 (0.003) | 0.64 (0.016) |

*Results from univariate mixed effects logistic regression with robust standard errors using indicator for missingness (0= not missing, 1= missing). Using all selected baseline variables in multivariate analysis, we found that only sex, BMI and age were statistically significant (p<0.05).

## **Table S4:** Logistic regression for missingness of outpatient visits and EQ-5D utility previous observed values*****

|  | **Missing data on outpatient visits** | **Missing data on EQ-5D** |
| --- | --- | --- |
| **Observed variables** | **p-value** | **p-value** |
| Previous year | 0.296 | 0.158 |
| Previous 1-2 years | 0.601 | 0.007 |
| Previous 1-3 years | 0.001 | 0.066 |
| Previous 1-4 years | 0.032 | 0.023 |

* Mixed effects logistic regression with robust standard errors using indicator for missingness (0= not missing, 1= missing) and lagged observed variable values. P-value estimated after testing equality of lagged variables and whether coefficients are 0.

## **Table S5:** Within Trial Resource Use by Year (following imputation of missing data)

|  | **Acarbose** | | | **Placebo** | | |
| --- | --- | --- | --- | --- | --- | --- |
|  | n | mean | (SD) | n | mean | (SD) |
| **Hospitalisations** |  |  |  |  |  |  |
| 12 months | 3,272 | 0.1 | (0.5) | 3,250 | 0.1 | (0.4) |
| 24 months | 3,190 | 0.1 | (0.4) | 3,156 | 0.1 | (0.4) |
| 36 months | 2,923 | 0.1 | (0.4) | 2,912 | 0.1 | (0.3) |
| 48 months | 2,666 | 0.1 | (0.3) | 2,644 | 0.1 | (0.3) |
| 60 months | 2,234 | 0.1 | (0.3) | 2,202 | 0.1 | (0.3) |
| 72 months | 1,623 | 0.1 | (0.3) | 1,604 | 0.1 | (0.3) |
| **Inpatient Days** |  |  |  |  |  |  |
| 12 months | 3,272 | 1.6 | (6.0) | 3,250 | 1.4 | (5.6) |
| 24 months | 3,190 | 1.1 | (5.2) | 3,156 | 1.1 | (4.8) |
| 36 months | 2,923 | 0.9 | (5.0) | 2,912 | 1.0 | (5.0) |
| 48 months | 2,666 | 0.7 | (3.5) | 2,644 | 0.7 | (4.2) |
| 60 months | 2,234 | 0.9 | (4.4) | 2,202 | 0.8 | (4.2) |
| 72 months | 1,623 | 0.8 | (5.0) | 1,604 | 0.7 | (3.7) |
| **Outpatient Visits** |  |  |  |  |  |  |
| 12 months | 3,272 | 11.0 | (13.4) | 3,250 | 10.9 | (13.0) |
| 24 months | 3,190 | 11.1 | (12.5) | 3,156 | 10.8 | (12.3) |
| 36 months | 2,923 | 10.4 | (14.0) | 2,912 | 10.3 | (13.3) |
| 48 months | 2,666 | 10.5 | (12.5) | 2,644 | 10.2 | (13.0) |
| 60 months | 2,234 | 10.4 | (13.8) | 2,202 | 10.5 | (13.8) |
| 72 months | 1,623 | 10.2 | (13.7) | 1,604 | 10.2 | (13.1) |
| **Diabetes Drug Days**^a^ |  |  |  |  |  |  |
| 12 months | 3,272 | 4.9 | (42.4) | 3,250 | 4.3 | (33.3) |
| 24 months | 3,190 | 10.4 | (59.1) | 3,156 | 11.7 | (57.0) |
| 36 months | 2,923 | 17.0 | (88.7) | 2,912 | 20.6 | (81.7) |
| 48 months | 2,666 | 19.8 | (90.6) | 2,644 | 27.7 | (96.1) |
| 60 months | 2,234 | 22.5 | (97.3) | 2,202 | 32.7 | (105.1) |
| 72 months | 1,623 | 22.4 | (84.9) | 1,604 | 31.9 | (98.2) |
| **CV Drug Days**^a^ |  |  |  |  |  |  |
| 12 months | 3,272 | 1,422 | (503) | 3,250 | 1,406 | (509) |
| 24 months | 3,190 | 1,321 | (524) | 3,156 | 1,333 | (535) |
| 36 months | 2,923 | 1,292 | (548) | 2,912 | 1,312 | (557) |
| 48 months | 2,666 | 1,229 | (576) | 2,644 | 1,238 | (584) |
| 60 months | 2,234 | 1,158 | (602) | 2,202 | 1,173 | (592) |
| 72 months | 1,623 | 980 | (604) | 1,604 | 1,004 | (604) |
| **Total Drug Days**^a^ |  |  |  |  |  |  |
| 12 months | 3,272 | 1,427 | (504) | 3,250 | 1,411 | (510) |
| 24 months | 3,190 | 1,332 | (527) | 3,156 | 1,345 | (537) |
| 36 months | 2,923 | 1,309 | (558) | 2,912 | 1,333 | (563) |
| 48 months | 2,666 | 1,249 | (588) | 2,644 | 1,266 | (594) |
| 60 months | 2,234 | 1,180 | (618) | 2,202 | 1,206 | (607) |
| 72 months | 1,623 | 1,002 | (612) | 1,604 | 1,036 | (622) |
| **Study Drug Days** |  |  |  |  |  |  |
| 12 months | 3,272 | 323 | (114) | 3,250 | - | - |
| 24 months | 3,190 | 311 | (122) | 3,156 | - | - |
| 36 months | 2,923 | 312 | (122) | 2,912 | - | - |
| 48 months | 2,666 | 303 | (124) | 2,644 | - | - |
| 60 months | 2,234 | 285 | (130) | 2,202 | - | - |
| 72 months | 1,623 | 244 | (135) | 1,604 | - | - |

^a^ Represents the number of drugs per day summed across the follow-up period for each patient excluding study drug. CV – cardiovascular, SD – standard deviation.

## **Table S5:** Within Trial Costs by Year (following imputation of missing data)

|  | **Acarbose** | | | **Placebo** | | | **Difference (Acarbose vs. placebo)**^c^ | |
| --- | --- | --- | --- | --- | --- | --- | --- | --- |
| Chinese Yuan (2017)^a^ | n | mean | (SD) | n | mean | (SD) | Mean ratio^d^ | p-value |
| **Hospitalisation Cost** |  |  |  |  |  |  |  |  |
| 12 months | 3,272 | ¥1,638 | (6,658) | 3,250 | ¥1,547 | (6,240) | 1.07 | 0.32 |
| 24 months | 3,190 | ¥1,166 | (5,608) | 3,156 | ¥1,185 | (5,471) | 0.99 | 0.92 |
| 36 months | 2,923 | ¥950 | (4,902) | 2,912 | ¥1,027 | (5,199) | 0.91 | 0.62 |
| 48 months | 2,666 | ¥652 | (3,351) | 2,644 | ¥753 | (4,030) | 0.87 | 0.32 |
| 60 months | 2,234 | ¥883 | (4,300) | 2,202 | ¥766 | (3,933) | 1.21 | 0.32 |
| 72 months | 1,623 | ¥817 | (4,615) | 1,604 | ¥643 | (3,482) | 1.28 | 0.14 |
| **Outpatient Cost** |  |  |  |  |  |  |  |  |
| 12 months | 3,272 | ¥3,995 | (4,735) | 3,250 | ¥3,908 | (4,464) | 1.01 | 0.49 |
| 24 months | 3,190 | ¥3,774 | (4,364) | 3,156 | ¥3,694 | (4,301) | 1.02 | 0.53 |
| 36 months | 2,923 | ¥3,453 | (4,718) | 2,912 | ¥3,422 | (4,505) | 1.01 | 0.83 |
| 48 months | 2,666 | ¥3,213 | (4,018) | 2,644 | ¥3,148 | (4,144) | 1.02 | 0.61 |
| 60 months | 2,234 | ¥2,920 | (4,167) | 2,202 | ¥2,988 | (4,285) | 0.97 | 0.54 |
| 72 months | 1,623 | ¥2,381 | (3,650) | 1,604 | ¥2,421 | (3,618) | 0.97 | 0.63 |
| **Diabetes Drug Cost**^b^ |  |  |  |  |  |  |  |  |
| 12 months | 3,272 | ¥25 | (403) | 3,250 | ¥19 | (321) | 1.40 | 0.42 |
| 24 months | 3,190 | ¥41 | (427) | 3,156 | ¥40 | (319) | 0.97 | 0.90 |
| 36 months | 2,923 | ¥63 | (659) | 2,912 | ¥66 | (442) | 0.93 | 0.81 |
| 48 months | 2,666 | ¥68 | (643) | 2,644 | ¥85 | (428) | 0.79 | 0.25 |
| 60 months | 2,234 | ¥72 | (634) | 2,202 | ¥104 | (545) | 0.68 | <0.001 |
| 72 months | 1,623 | ¥65 | (383) | 1,604 | ¥101 | (510) | 0.63 | <0.001 |
| **CV Drug Cost** |  |  |  |  |  |  |  |  |
| 12 months | 3,272 | ¥4,397 | (2,180) | 3,250 | ¥4,365 | (2,212) | 1.01 | 0.57 |
| 24 months | 3,190 | ¥3,776 | (2,043) | 3,156 | ¥3,816 | (2,071) | 0.99 | 0.15 |
| 36 months | 2,923 | ¥3,534 | (2,008) | 2,912 | ¥3,599 | (2,031) | 0.98 | 0.24 |
| 48 months | 2,666 | ¥3,265 | (1,978) | 2,644 | ¥3,289 | (1,990) | 0.99 | 0.65 |
| 60 months | 2,234 | ¥2,956 | (1,907) | 2,202 | ¥2,978 | (1,882) | 0.99 | 0.64 |
| 72 months | 1,623 | ¥2,384 | (1,708) | 1,604 | ¥2,445 | (1,746) | 0.97 | 0.35 |
| **Total drug cost**^b^ |  |  |  |  |  |  |  |  |
| 12 months | 3,272 | ¥4,422 | (2,214) | 3,250 | ¥4,385 | (2,223) | 1.01 | 0.46 |
| 24 months | 3,190 | ¥3,817 | (2,106) | 3,156 | ¥3,856 | (2,085) | 0.99 | 0.18 |
| 36 months | 2,923 | ¥3,597 | (2,187) | 2,912 | ¥3,665 | (2,073) | 0.98 | 0.29 |
| 48 months | 2,666 | ¥3,333 | (2,158) | 2,644 | ¥3,374 | (2,036) | 0.99 | 0.50 |
| 60 months | 2,234 | ¥3,028 | (2,105) | 2,202 | ¥3,083 | (1,962) | 0.98 | 0.30 |
| 72 months | 1,623 | ¥2,448 | (1,780) | 1,604 | ¥2,546 | (1,834) | 0.96 | 0.13 |
| **Study Drug Cost** |  |  |  |  |  |  |  |  |
| 12 months | 3,272 | ¥1,960 | (694) | 3,250 | - | - |  |  |
| 24 months | 3,190 | ¥1,864 | (734) | 3,156 | - | - |  |  |
| 36 months | 2,923 | ¥1,816 | (710) | 2,912 | - | - |  |  |
| 48 months | 2,666 | ¥1,716 | (699) | 2,644 | - | - |  |  |
| 60 months | 2,234 | ¥1,566 | (715) | 2,202 | - | - |  |  |
| 72 months | 1,623 | ¥1,302 | (719) | 1,604 | - | - |  |  |
| **Total cost**^b^ |  |  |  |  |  |  |  |  |
| 12 months | 3,272 | ¥10,062 | (9,003) | 3,250 | ¥9,885 | (8,580) | 1.01 | 0.43 |
| 24 months | 3,190 | ¥8,792 | (7,840) | 3,156 | ¥8,770 | (7857) | 1.00 | 0.90 |
| 36 months | 2,923 | ¥8,028 | (7,625) | 2,912 | ¥8,145 | (7,639) | 0.99 | 0.75 |
| 48 months | 2,666 | ¥7,217 | (6,181) | 2,644 | ¥7,297 | (6,462) | 0.98 | 0.51 |
| 60 months | 2,234 | ¥6,858 | (6,876) | 2,202 | ¥6,860 | (6,484) | 1.00 | 0.85 |
| 72 months | 1,623 | ¥5,671 | (6,632) | 1,604 | ¥5,628 | (5,853) | 1.01 | 0.84 |
| **Total cost** |  |  |  |  |  |  |  |  |
| 12 months | 3,272 | ¥12,023 | (9,035) | 3,250 | ¥9,885 | (8,580) | 1.22 | <0.001 |
| 24 months | 3,190 | ¥10,656 | (7,860) | 3,156 | ¥8,770 | (7,857) | 1.22 | <0.001 |
| 36 months | 2,923 | ¥9,844 | (7,688) | 2,912 | ¥8,145 | (7,639) | 1.22 | <0.001 |
| 48 months | 2,666 | ¥8,933 | (6,316) | 2,644 | ¥7,297 | (6,462) | 1.23 | <0.001 |
| 60 months | 2,234 | ¥8,424 | (7,027) | 2,202 | ¥6,860 | (6,484) | 1.23 | <0.001 |
| 72 months | 1,623 | ¥6,973 | (6,827) | 1,604 | ¥5,629 | (5,853) | 1.25 | <0.001 |

^a^ ¥1 = US$0.16; ¥1 = €0.13; ¥1 = £0.11.

^b^ Cost per patient excluding study drug.

^c^ Differences estimated using Stata MEGLM (multilevel mixed-effects generalized linear model) procedure specified at region level with log-link function and gamma distribution.

^d^ A mean ratio above one indicates a higher average cost in the treatment group relative to the placebo group, e.g. being in the treatment group increases the hospitalisation costs in year 1 by 7% relative to the placebo group.

CV – cardiovascular, SD – standard deviation.

## **Table S6:** Within Trial Costs by Year (using only observed data)

|  | **Acarbose** | | | **Placebo** | | | **Difference (Acarbose vs. placebo)**^c^ | |
| --- | --- | --- | --- | --- | --- | --- | --- | --- |
| Chinese Yuan (2017)^a^ | n | mean | (SD) | n | mean | (SD) | Mean ratio^d^ | p-value |
| **Hospitalisation Cost** |  |  |  |  |  |  |  |  |
| 12 months | 3,246 | ¥1,520 | (6,362) | 3,236 | ¥1,511 | (6,179) | 1.02 | 0.80 |
| 24 months | 3,164 | ¥1,077 | (5,294) | 3,143 | ¥1,205 | (5,739) | 0.91 | 0.26 |
| 36 months | 2,904 | ¥942 | (5,190) | 2,902 | ¥1,090 | (5,663) | 0.85 | 0.43 |
| 48 months | 2,652 | ¥661 | (3,612) | 2,630 | ¥762 | (4,313) | 0.87 | 0.33 |
| 60 months | 2,222 | ¥923 | (4,577) | 2,191 | ¥837 | (4,502) | 1.16 | 0.49 |
| 72 months | 1,618 | ¥925 | (5,422) | 1,599 | ¥726 | (4,082) | 1.28 | 0.09 |
| **Outpatient Cost** |  |  |  |  |  |  |  |  |
| 12 months | 2,299 | ¥3,918 | (4,014) | 2,226 | ¥3,871 | (3,859) | 1.01 | 0.28 |
| 24 months | 2,068 | ¥3,726 | (3,875) | 2,032 | ¥3,672 | (3,835) | 1.02 | 0.42 |
| 36 months | 1,781 | ¥3,437 | (3,646) | 1,705 | ¥3,544 | (3,785) | 0.98 | 0.50 |
| 48 months | 1,489 | ¥3,145 | (3,349) | 1,462 | ¥3,199 | (3,453) | 0.99 | 0.76 |
| 60 months | 1,170 | ¥2,900 | (3,202) | 1,142 | ¥2,963 | (3,481) | 0.97 | 0.35 |
| 72 months | 817 | ¥2,441 | (3,053) | 810 | ¥2,536 | (3,155) | 0.98 | 0.69 |
| **Diabetes Drug Cost**^b^ |  |  |  |  |  |  |  |  |
| 12 months | 2,623 | ¥31 | (450) | 2,579 | ¥14 | (98) | 2.24 | 0.01 |
| 24 months | 2,248 | ¥49 | (410) | 2,201 | ¥47 | (234) | 0.98 | 0.95 |
| 36 months | 1,912 | ¥62 | (305) | 1,856 | ¥87 | (447) | 0.71 | 0.08 |
| 48 months | 1,462 | ¥75 | (315) | 1,450 | ¥119 | (437) | 0.63 | <0.001 |
| 60 months | 1,043 | ¥84 | (330) | 1,011 | ¥143 | (492) | 0.59 | <0.001 |
| 72 months | 524 | ¥94 | (386) | 516 | ¥117 | (333) | 0.81 | 0.18 |
| **CV Drug Cost** |  |  |  |  |  |  |  |  |
| 12 months | 2,621 | ¥4,041 | (2,009) | 2,576 | ¥4,017 | (2,024) | 1.01 | 0.59 |
| 24 months | 2,247 | ¥3,422 | (1,799) | 2,202 | ¥3,443 | (1,816) | 0.99 | 0.39 |
| 36 months | 1,911 | ¥3,192 | (1,719) | 1,855 | ¥3,267 | (1,739) | 0.98 | 0.23 |
| 48 months | 1,463 | ¥3,122 | (1,666) | 1,449 | ¥3,077 | (1,626) | 1.02 | 0.37 |
| 60 months | 1,043 | ¥2,961 | (1,548) | 1,010 | ¥2,948 | (1,535) | 1.01 | 0.82 |
| 72 months | 524 | ¥2,710 | (1,396) | 515 | ¥2,732 | (1,454) | 0.99 | 0.67 |
| **Total drug cost**^b^ |  |  |  |  |  |  |  |  |
| 12 months | 2,621 | ¥4,193 | (2,118) | 2,576 | ¥4,151 | (2,083) | 1.01 | 0.25 |
| 24 months | 2,247 | ¥3,564 | (1,925) | 2,202 | ¥3,585 | (1,875) | 0.99 | 0.44 |
| 36 months | 1,911 | ¥3,342 | (1,785) | 1,855 | ¥3,440 | (1,846) | 0.98 | 0.23 |
| 48 months | 1,463 | ¥3,280 | (1,729) | 1,449 | ¥3,270 | (1,718) | 1.00 | 0.90 |
| 60 months | 1,043 | ¥3,104 | (1,622) | 1,010 | ¥3,148 | (1,658) | 0.99 | 0.78 |
| 72 months | 524 | ¥2,802 | (1,458) | 515 | ¥2,862 | (1,533) | 0.98 | 0.56 |
| **Study Drug Cost** |  |  |  |  |  |  |  |  |
| 12 months | 2,653 | ¥1,933 | (737) |  |  |  |  |  |
| 24 months | 2,286 | ¥1,841 | (799) |  |  |  |  |  |
| 36 months | 1,941 | ¥1,782 | (782) |  |  |  |  |  |
| 48 months | 1,478 | ¥1,743 | (745) |  |  |  |  |  |
| 60 months | 1,049 | ¥1,624 | (775) |  |  |  |  |  |
| 72 months | 524 | ¥1,475 | (804) |  |  |  |  |  |
| **Total cost**^b^ |  |  |  |  |  |  |  |  |
| 12 months | 2280 | ¥9,448 | (7,340) | 2,217 | ¥9,430 | (7,649) | 1.00 | 0.96 |
| 24 months | 1940 | ¥8,425 | (6,693) | 1,908 | ¥8,581 | (7,368) | 0.99 | 0.42 |
| 36 months | 1657 | ¥7,798 | (6,881) | 1,599 | ¥8,248 | (7,631) | 0.96 | 0.27 |
| 48 months | 1277 | ¥7,340 | (5,567) | 1,280 | ¥7,608 | (6,134) | 0.95 | 0.19 |
| 60 months | 904 | ¥7,321 | (6,596) | 885 | ¥7,591 | (6,292) | 0.95 | 0.26 |
| 72 months | 479 | ¥7,090 | (6,863) | 473 | ¥7,132 | (6,396) | 0.98 | 0.76 |
| **Total cost** |  |  |  |  |  |  |  |  |
| 12 months | 2280 | ¥11,442 | (7,354) | 2,217 | ¥9,430 | (7,649) | 1.22 | <0.001 |
| 24 months | 1940 | ¥10,342 | (6,698) | 1,908 | ¥8,581 | (7,368) | 1.22 | <0.001 |
| 36 months | 1657 | ¥9,649 | (6,900) | 1,599 | ¥8,248 | (7,631) | 1.20 | <0.001 |
| 48 months | 1277 | ¥9,095 | (5,590) | 1,280 | ¥7,340 | (5,567) | 1.19 | <0.001 |
| 60 months | 904 | ¥8,978 | (6,624) | 885 | ¥7,591 | (6,292) | 1.18 | <0.001 |
| 72 months | 479 | ¥8,568 | (6,859) | 473 | ¥7,132 | (6,396) | 1.20 | <0.001 |

^a^ ¥1 = US$0.16; ¥1 = €0.13; ¥1 = £0.11.

^b^ Cost per patient excluding study drug.

^c^ Differences estimated using Stata MEGLM (multilevel mixed-effects generalized linear model) procedure specified at region level with log-link function and gamma distribution.

^d^ A mean ratio above one indicates a higher average cost in the treatment group relative to the placebo group, e.g. being in the treatment group increases the hospitalisation costs in year 1 by 2% relative to the placebo group.

CV – cardiovascular, SD – standard deviation.

## **Table S7:** Sensitivity Analysis on between group differences in within trial costs when varying discount rates between 1% and 5% (following imputation of missing data)

|  | **Difference (acarbose vs. placebo)** | | | | | |
| --- | --- | --- | --- | --- | --- | --- |
|  | **3% (base case)** | | **1%** | | **5%** | |
| Cost category | Mean ratio^b^ | (p-value)^c^ | Mean ratio^b^ | (p-value)^c^ | Mean ratio^b^ | (p-value)^c^ |
| Inpatient Care Cost | 1.02 | (0.60) | 1.02 | (0.38) | 1.02 | (0.41) |
| Outpatient Care Cost | 1.01 | (0.49) | 1.01 | (0.52) | 1.01 | (0.49) |
| Diabetes Drug Cost^a^ | 0.84 | (0.23) | 0.83 | (0.21) | 0.84 | (0.25) |
| CV Drug Cost | 0.99 | (0.30) | 0.99 | (0.71) | 0.99 | (0.71) |
| Total Drugs Cost^a^ | 0.99 | (0.60) | 0.99 | (0.60) | 0.99 | (0.61) |
| Study Drug Cost | - | - |  |  |  |  |
| Total Cost^a^ | 1.00 | (0.89) | 1.00 | (0.90) | 1.00 | (0.79) |
| Total Cost | 1.23 | (<0.001) | 1.18 | (<0.001) | 1.18 | (<0.001) |

^a^ Cost per patient excluding study drug.

^b^ A mean ratio above one indicates a higher average cost in the treatment group.

^c^ p-value from Stata MEGLM procedure specified at region level, log-link function and gamma distribution.

CV – cardiovascular, SD – standard deviation.

## **Figure S1**: Cost-effectiveness scatter plot (within trial)

##
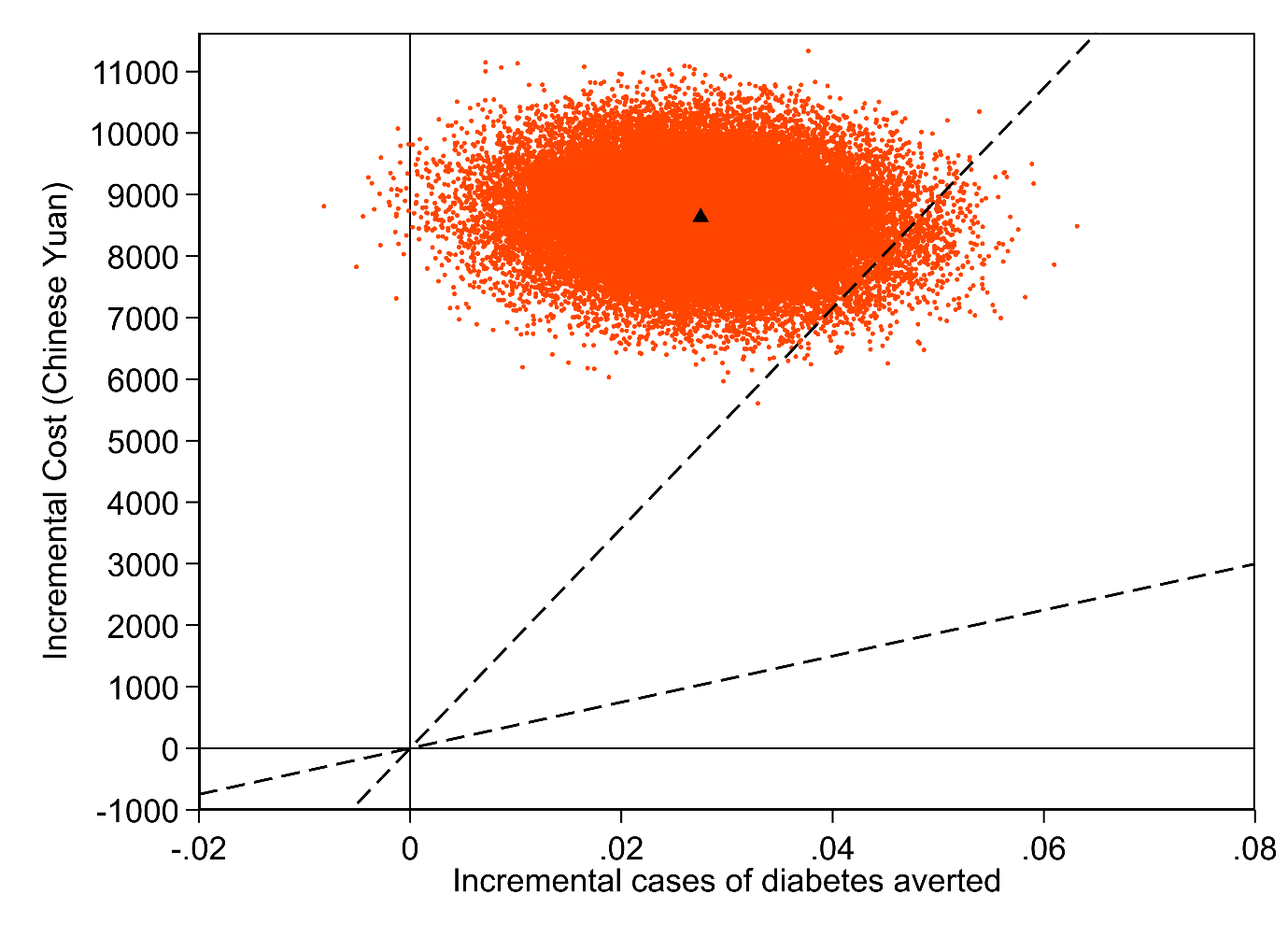


¥178,980/diabetes averted

¥37,446/diabetes averted

*Scatter plot of estimated joint density of incremental costs and cases of diabetes averted of acarbose relative to placebo obtained by bootstrap re-sampling from each of the imputed datasets, running the regression models on each bootstrapped dataset and extracting the estimated incremental costs and cases of diabetes averted. The black triangle represents the estimated mean incremental costs and diabetes cases averted. Dashed lines represent threshold values of ¥37,446*^21^ *and ¥178,980 (3x GDP per capita*^22^*) per diabetes case averted. Bootstrapped results falling below the lines are deemed cost-effective.*
